# Supplementary material for: Multimodal BEHRT: transformers for multimodal electronic health records to predict breast cancer prognosis
Source: Front Oncol. 2025 Oct 17;15:1496215. doi: 10.3389/fonc.2025.1496215 (PMC12575146; doi:10.3389/fonc.2025.1496215)
Supplement: Supplementary file 1 [file DataSheet1.pdf]

Supplementary Text to Multimodal BEHRT: Transformers for Multimodal Electronical Health Records

1 PREPROCESSING OF FREE TEXT

Removing proper nouns is one of the key step of the preprocessing pipeline. This is important as specific doctor names may serve as proxy for the DFS classification, for example, when a doctor mostly handles severe cases. Patient names are already excluded from the reports, which had been anonymized before we accessed them. The first stage of this process consists in using part-of-speech tagging to remove proper nouns tags that follow titles such as *Dr*, *M.* (“Mr” in English), *Mme* (“Mrs” in English). However, proper nouns may appear without a title. We thus further constructed a list of proper nouns to remove from the text. We first built a list of names of Institut Curie’s health practicioners, obtained through the public directory of practicioners Cur (Accessed: 2023-01-30) as retrieved in 2023, and therefore only partially matching practicioners that were involved in the care of patients in the 2005–2012 period covered by our cohort). We additionally considered surnames given at least 30 times in France from 1891 to 2000 (n=218 912) and first names given at least 20 times from 1946 to 2022 in France (n=36 964), as provided by Institut National de La Statistique et des Etudes Econonomiques (INSEE) (Ins (Accessed: 2023-01-30), INS (Accessed: 2023-01-30)). We then removed from this list the proper names that correspond to disease names, such as Paget.

One other main difficulty that occur with free-text reports is the high number of typos. To address this issue, we used the pyspellchecker spell checking algorithm Barus (2023) which identifies, for each word of the corpus that is not found in a given dictionary, the most likely correct replacement for this presumably misspelled word. For effective spellchecking, it is crucial to have a rich dictionary that contains medical jargon. Therefore, we augmented the French vocabulary from OpenSubtitles Lison and Tiedemann (2016) (implemented by default in pyspellchecker) with the contents of the French open dictionary Usito ush (Accessed: 2023-01-30), as well as the 3 184 words from a French online medical dictionary Thomsen (Accessed: 2023-01-30), the CAS corpus of French clinical cases Grabar et al. (2018) which contains over 397 000 word occurences, a list of drug names in French vid (Accessed: 2023-01-30), and two lists of French medical abbreviations specific to oncology moz (2020); Poletto (2023). If, following this step, any words from the dictionary remain unidentified, we replaced them with the most likely correct spelling suggestion from Wikipedia wik (Accessed: 2023-01-30).

2 TEXT BEHRT INTERPRETATION

We choose to analyze the most frequent sequences for the DFS negative cohort that are not found in the DFS positive cohort. We ended up with the following sequences of words, some of which have been obtained with the overlapping resulting sequences:

- “sein en involution adipeuse partielle avec contingent glandulaire inferieur a 50”, (*breast in partial adipose involution with less than 50% glandular contingent*)
- “Traitement anterieur par hormone de croissance extractible non facteurs de risque de transmission de la mcj”, (*Previous treatment with extractable growth hormone without risk factors for mcj transmission*)

- "[avec] lymphadenectomie axillaire", (*with axillary lymphadenectomy*)
- "syndrome de masse", (*mass syndrom*)
- "[j1] solumedrol 80mg", (*solumedrol 80mg*)
- "lovenox 0 4 ml", (*lovenox 0 4 ml*)

REFERENCES

Curie - annuaire 2023 (Accessed: 2023-01-30). https://curie.fr/annuaire-medecins.

Insee noms (Accessed: 2023-01-30). https://www.insee.fr/fr/statistiques/3536630.

Insee prenoms (Accessed: 2023-01-30). https://www.insee.fr/fr/statistiques/7633685?sommaire=7635552.

Barus T. pyspellchecker – Pure python spell checker based on work by Peter Norvig (2023). https://pypi.org/project/pyspellchecker.

Lison P, Tiedemann J. OpenSubtitles2016: Extracting large parallel corpora from movie and TV subtitles. Calzolari N, Choukri K, Declerck T, Goggi S, Grobelnik M, Maegaard B, et al., editors, *Proceedings of the Tenth International Conference on Language Resources and Evaluation (LREC'16)* (Portorož, Slovenia: European Language Resources Association (ELRA)) (2016), 923–929.

Usito, dictionnaire général de la langue française (Accessed: 2023-01-30). Université de Sherbrooke https://usito.usherbrooke.ca/.

Thomsen C. Dictionnaire Médical (Accessed: 2023-01-30). https://www.dictionnaire-medical.fr/.

Grabar N, Claveau V, Dalloux C. CAS: French corpus with clinical cases. Lavelli A, Minard AL, Rinaldi F, editors, *Proceedings of the Ninth International Workshop on Health Text Mining and Information Analysis* (Brussels, Belgium: Association for Computational Linguistics) (2018), 122–128. doi:10.18653/v1/W18-5614.

Le Dictionnaire VIDAL (Accessed: 2023-01-30). https://www.vidal.fr/medicaments.html.

Oncopod – abréviations pour l'oncologie (2020). https://www.mozocare.com/fr/oncopod/chemotherapy/abbreviations/ (Accessed: 2023-01-30), Mozocare.

Poletto B. Glossaire info cancer (2023). https://www.arcagy.org/infocancer/cms/glossaire, (Accessed: 2023-01-30).

Wikipédia – l'encyclopédie libre (Accessed: 2023-01-30). https://fr.wikipedia.org.
