# Supplementary material for: Multimodal BEHRT: transformers for multimodal electronic health records to predict breast cancer prognosis
Source: Front Oncol. 2025 Oct 17;15:1496215. doi: 10.3389/fonc.2025.1496215 (PMC12575146; doi:10.3389/fonc.2025.1496215)
Supplement: Supplementary file 2 [file DataSheet2.pdf]

Supplementary Material to Multimodal BEHRT: Transformers for Multimodal Electronical Health Records

1 SUPPLEMENTARY TABLES AND FIGURES

Feature	Normal range	Mean value ± std	missing
CA15-3 (U/ml)	$N < 30$	63.39 ± 484.44	6 390
LEUK (g/l)	$4 < N < 10$	6.99 ± 6.82	2 525
PN (g/l)	$1.7 < N < 7$	$718.85 \pm 1\,789.66$	9 419
LYMP (g/l)	$1.4 < N < 4$	289.63 ± 714.26	9 448
MONO (g/l)	$0.2 < N < 1$	33.29 ± 123.59	3 675

Table S1. Normal ranges for the biological features

Features		Entire dataset		Dataset for DFS at 3 years	
		Mean ± std	N	Mean ± std	N
Age	< 50	58 ± 12	3 982	56 ± 12	2 493
	≥ 50		11 168		5 596
BC subtype	Luminal		9 979		4 866
	TNBC		1 041		642
	HER2+/HR+		681		587
	HER2+/HR-		480		415
Grades	I		3 473		1 688
	II		5 911		3 057
	III		3 119		2 044
Nodes	N0	0.93 ± 2.49	9 463	1.07 ± 2.74	4 899
	N+		4 045		2 405
Tumor size (mm)	Clinical	16.89 ± 12.70		17.36 ± 12.97	
	Pathological	15.04 ± 12.75		15.63 ± 12.90	
Biological values	CA 15-3 (U/ml)	63.39 ± 484.44	8 760	62.85 ± 535.76	3 826
	LEUK (g/l)	6.99 ± 6.82	12 625	6.90 ± 7.49	6 419
	PN (g/l)	718.85 ± 1789.66	5 731	976.17 ± 2007.52	2 385
	LYMP (g/l)	289.63 ± 714.26	5 702	405.84 ± 820.08	2 373
	MONO (g/l)	33.29 ± 123.59	11 475	37.54 ± 131.79	5 821
Medical reports	visits	46 ± 33		25 ± 10	
	reports	62 ± 50		34 ± 15	
	words/report	172 ± 41		159 ± 37	

Table S2. Descriptive statistics of the data used in this study, for the full cohort of 15 150 patients, as well as the data set of patients uncensored 3 years after surgery.

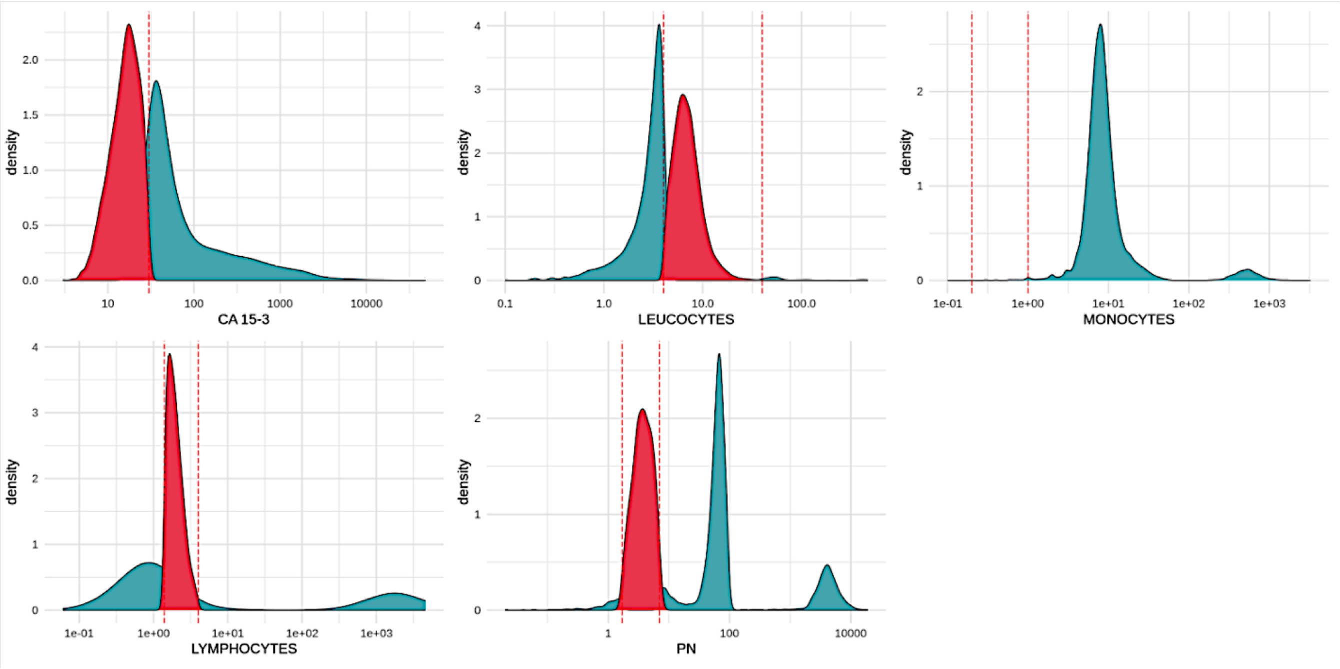

Figure S1. Binarization of biological features into two values, 1 and 2. For each of the 5 biological features, the dashed red lines delineate the normal range, highlighted in red, and mapped to 2, from the abnormal range, highlighted in green, and mapped to 1

Therapies	Sub-therapies
Surgery	Lumpectomy<br>Mastectomy<br>Axillary node dissection<br>Sentinel node biopsy
Radiotherapy	Axillary irradiation<br>Internal mammary chain irradiation<br>Mammary gland/chest wall irradiation<br>Supra/sub-clavicular irradiation
Hormone therapy	Tamoxifen<br>Aromatase<br>LHRH agonist
Anti-HER2 therapy	Trastuzumab<br>Pertuzumab<br>Lapatinib

Table S3. List of possible therapies and sub-therapies in our data.

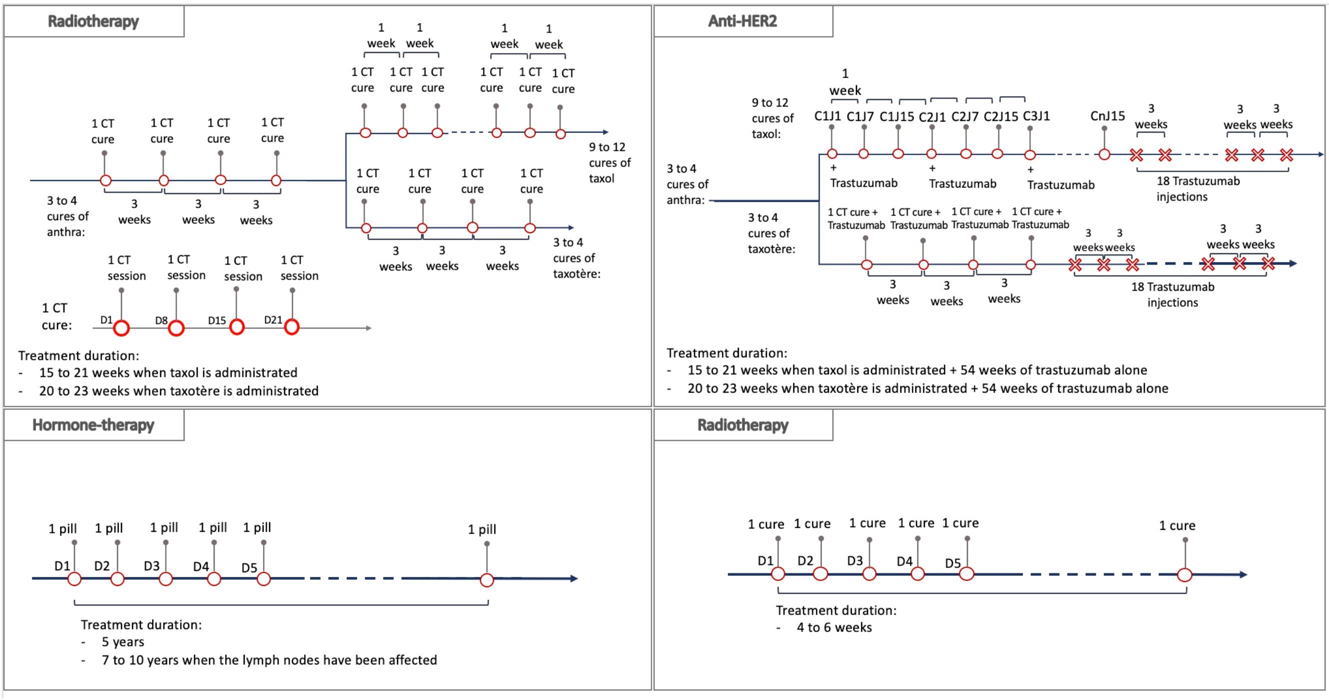

Figure S2. Institut Curie Therapeutic Protocol

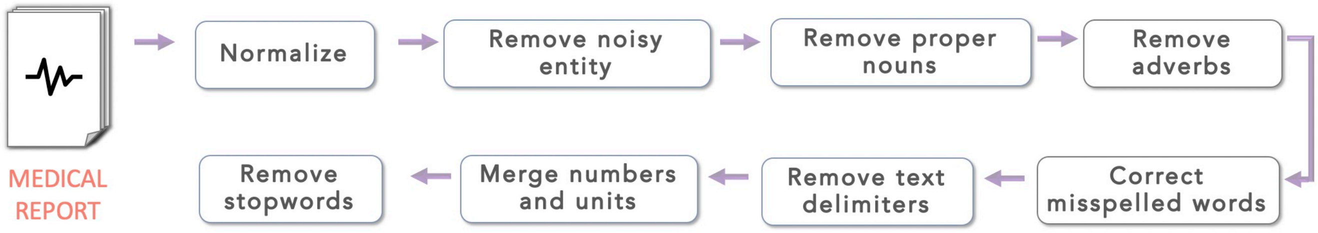

Figure S3. Text preprocessing pipeline.

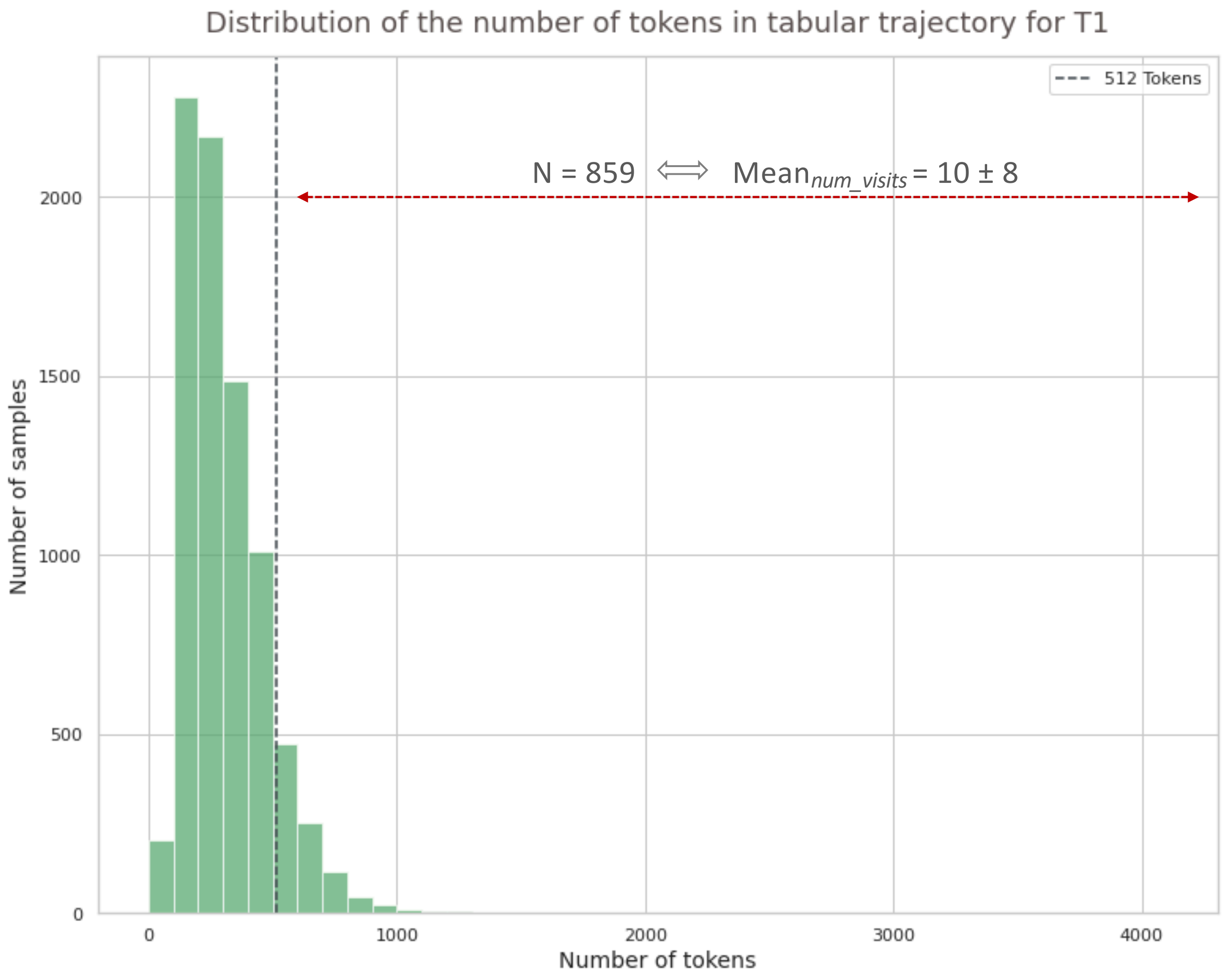

Figure S4. Distribution of the number of tokens per patient trajectory, for the prediction of disease-free survival 3 years after surgery. 859 samples exceed the maximum sequence length for Tabular BEHRT (512 tokens). This represents an average of 10 visits per patient that are not considered by Tabular BEHRT.

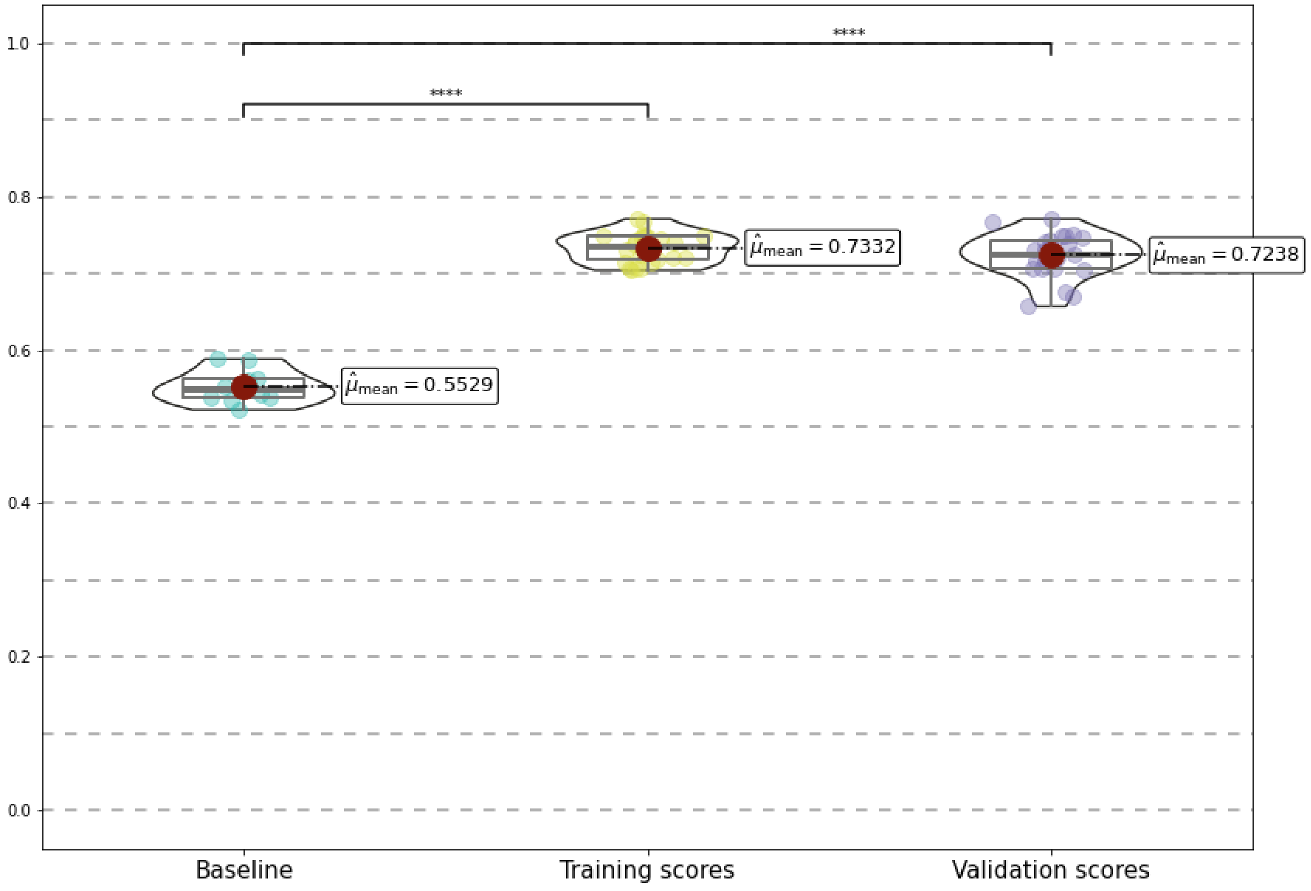

Figure S5. Precision scores for the Masked Language Model (pre-training of Tabular BEHRT). The baseline scores are obtained from the MLM ran on shuffled sequences.

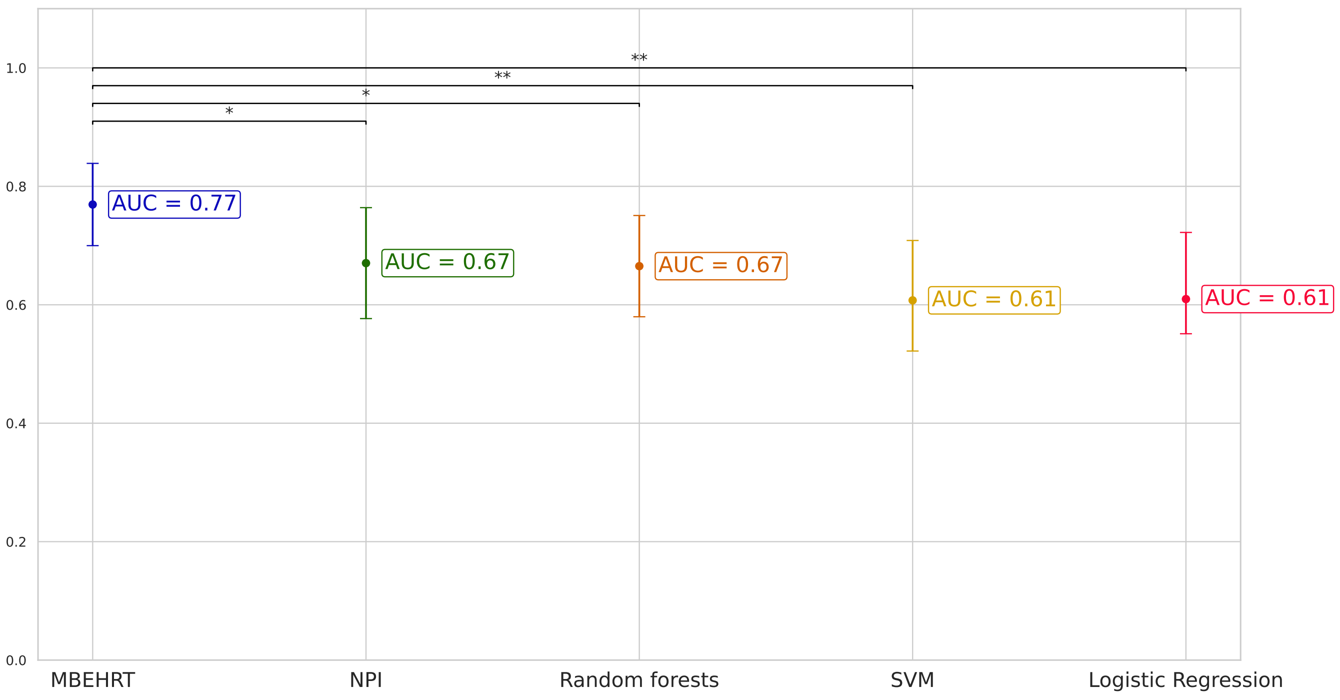

Figure S6. AUC scores comparison between M-BEHRT and the baselines for the prediction of disease-free survival 3 years after the surgery on the test set. * indicates a p-value lower than 0.05 and ** a p-value lower than 0.01. p-values are computed with the DeLong statistical test.

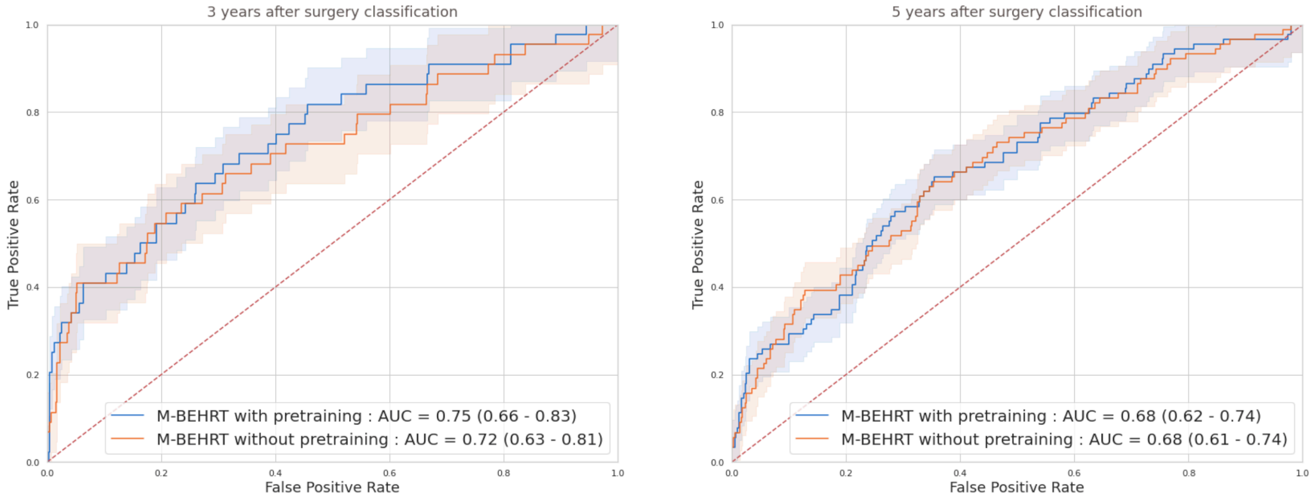

Figure S7. ROC curves on the test set of Tabular BEHRT, with and without pre-training, for the prediction of disease-free survival 3 (left) or 5 (right) years after surgery.

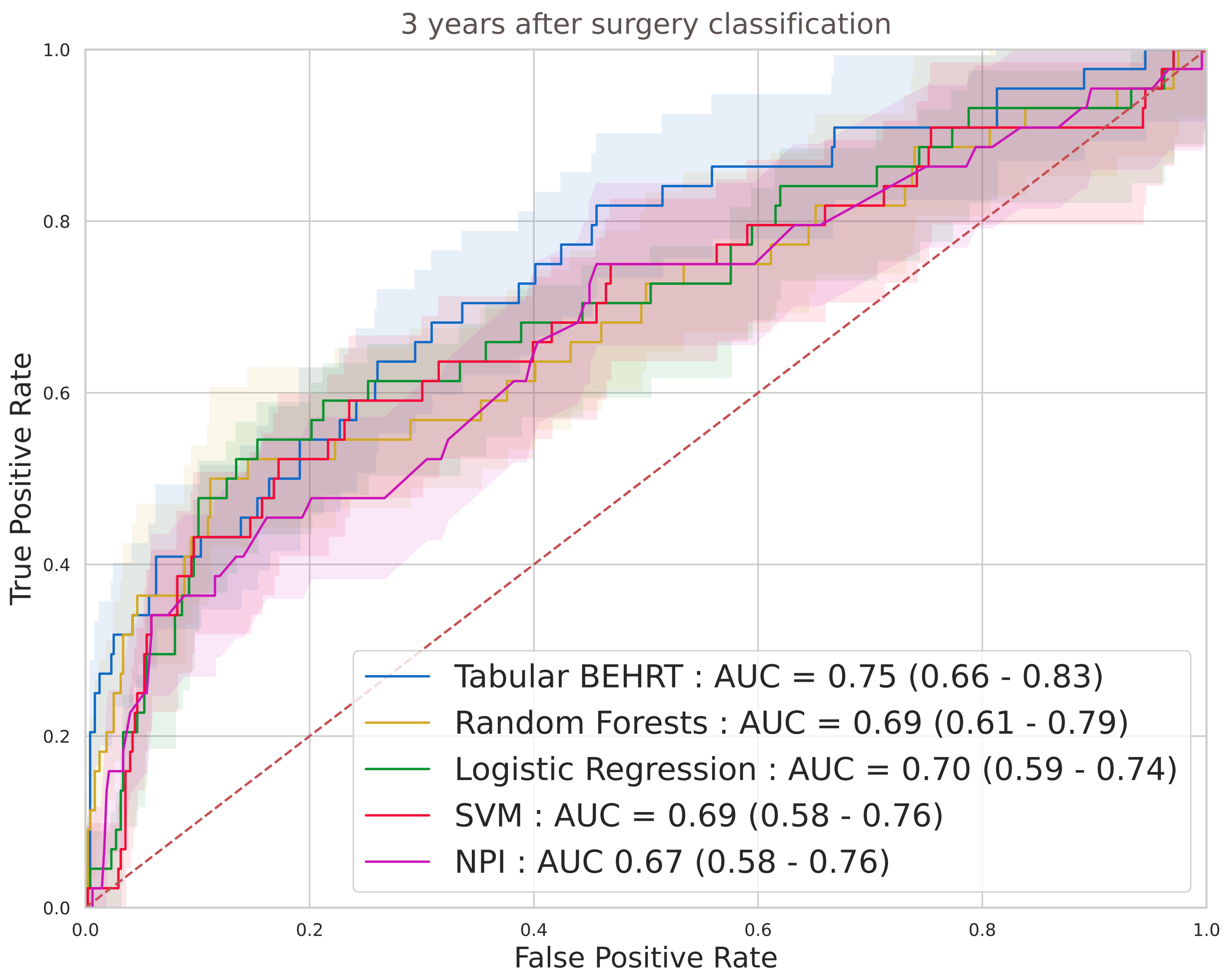

Figure S8. ROC curves for baselines and Tabular BEHRT, for predicting disease-free survival 3 years after surgery.

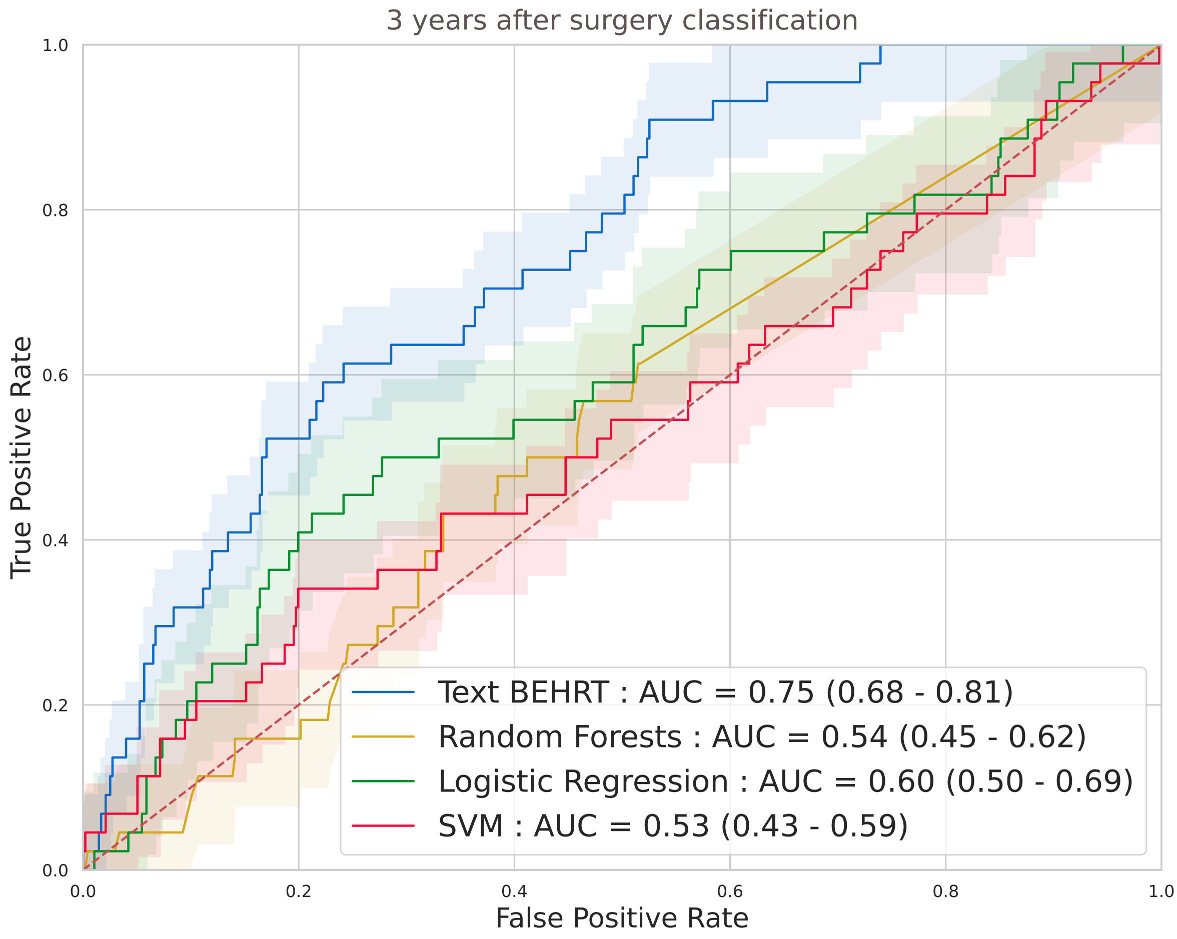

Figure S9. ROC curves for baselines and Text BEHRT, for predicting disease-free survival 3 years after surgery.

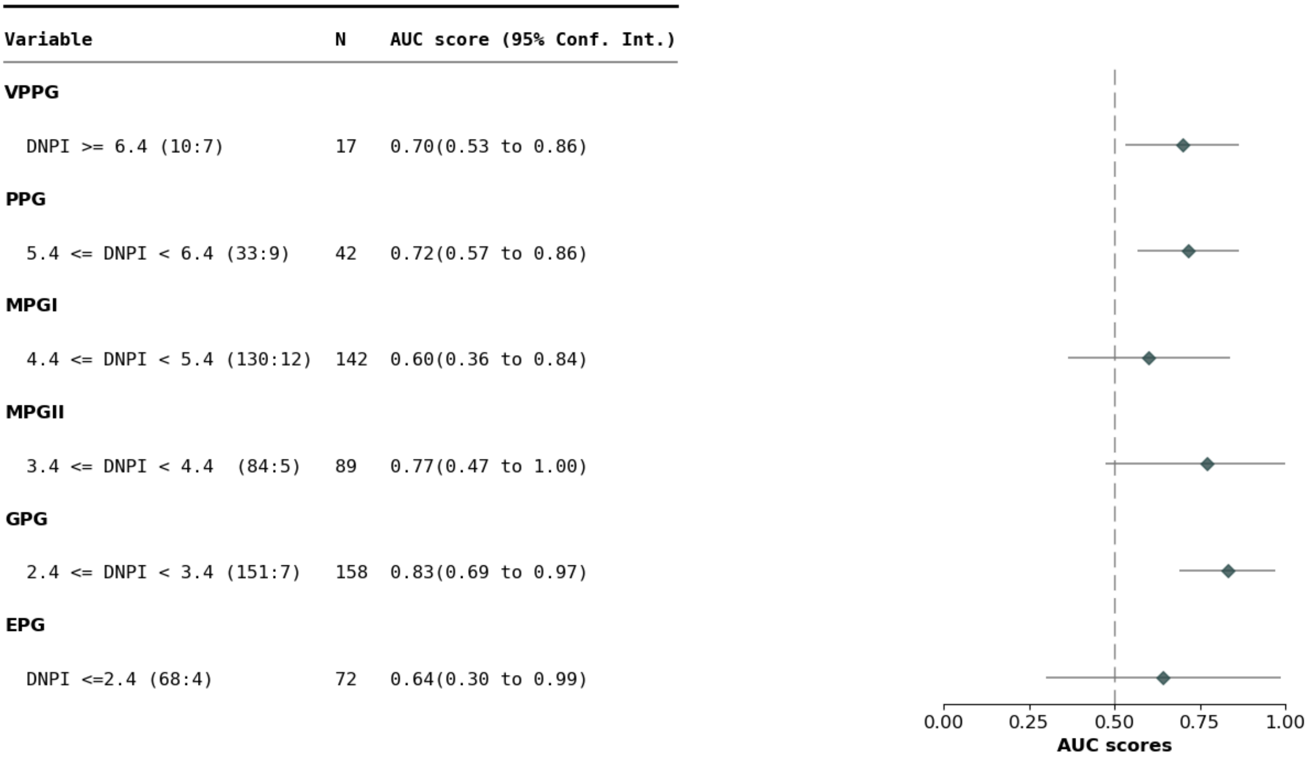

Figure S10. AUC-ROC of M-BEHRT stratified by NPI, for predicting disease-free survival 3 years after surgery.

Features		Entire dataset		Dataset for DFS at 3 years		Dataset for DFS at 5 years	
		Mean ± std	N	Mean ± std	N	Mean ± std	N
Age	< 50	58 ± 12	3 982	56 ± 12	2 493	55 ± 13	1 725
	≥ 50		11 168		5 596		3 467
BC subtype	Luminal		9 979		4 866		2 930
	TNBC		1 041		642		446
	HER2+/HR+		681		587		482
	HER2+/HR-		480		415		330
Grades	I		3 473		1 688		1 016
	II		5 911		3 057		1 941
	III		3 119		2 044		1 462
Nodes	N0	0.93 ± 2.49	9 463	1.07 ± 2.74	4 899	1.18 ± 3.01	3 132
	N+		4 045		2 405		1 597
Tumor size (mm)	Clinical	16.89 ± 12.70		17.36 ± 12.97		17.78 ± 13.18	
	Pathological	15.04 ± 12.75		15.63 ± 12.90		16.17 ± 12.94	
Biological values	CA 15-3 (U/ml)	63.39 ± 484.44	8 760	62.85 ± 535.76	3 826	75.34 ± 617.09	2 256
	LEUK (g/l)	6.99 ± 6.82	12 625	6.90 ± 7.49	6 419	6.75 ± 3.60	3 916
	PN (g/l)	718.85 ± 1789.66	5 731	976.17 ± 2007.52	2 385	1105.76 ± 2093.81	1 365
	LYMP (g/l)	289.63 ± 714.26	5 702	405.84 ± 820.08	2 373	463.92 ± 862.37	1 375
	MONO (g/l)	33.29 ± 123.59	11 475	37.54 ± 131.79	5 821	33.29 ± 123.59	3 489
Medical reports	visits	46 ± 33		25 ± 10		25 ± 10	
	reports	62 ± 50		34 ± 15		34 ± 15	
	words/report	172 ± 41		159 ± 37		159 ± 37	

Table S4. Descriptive statistics of the data sets used in this study, for the full cohort of 15 150 patients, as well as the data set of patients uncensored 3 years and 5 years after surgery.

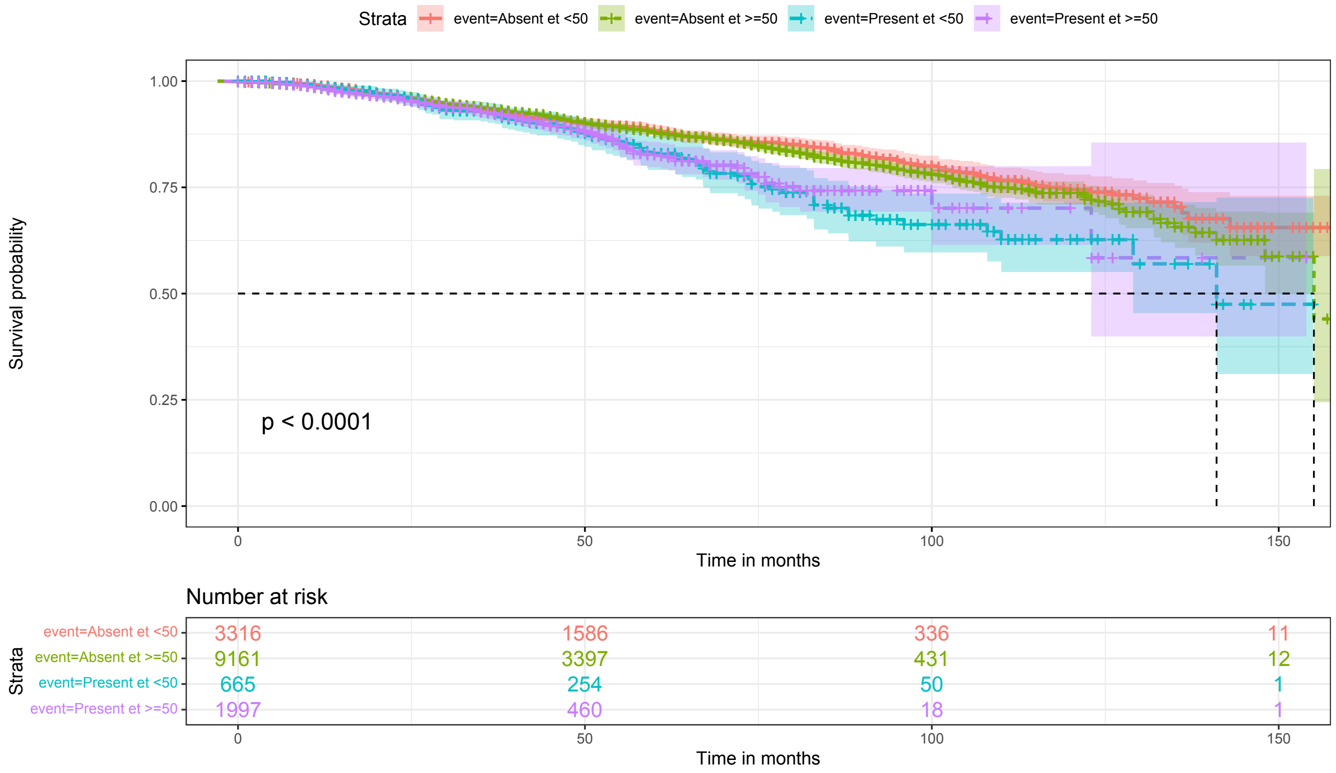

Figure S11. Survival plots for the presence/absence of the sentence meaning "breast in partial involution with less than 50% glandular tissue", combined with the feature "age" (> 50 vs < 50).

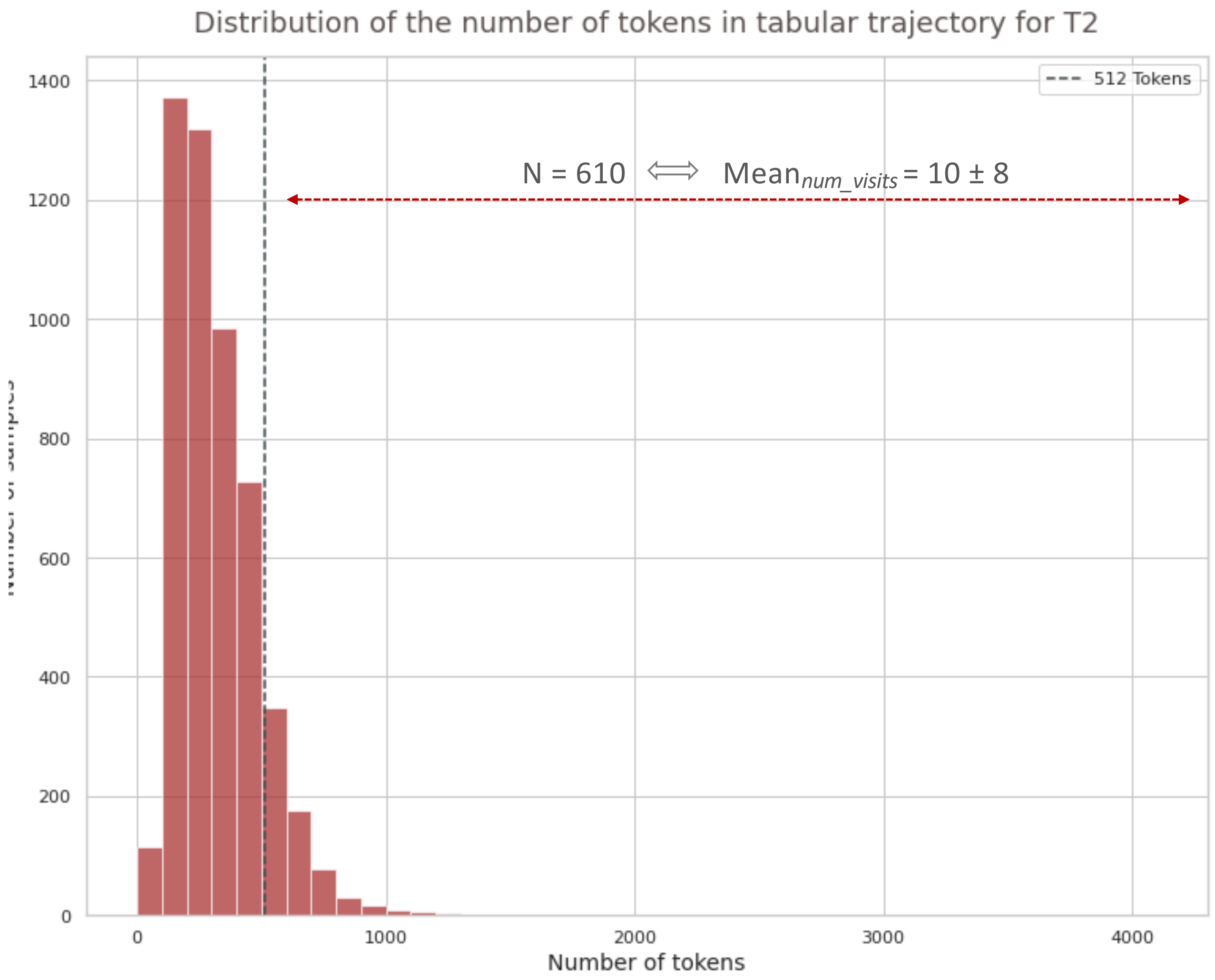

Figure S12. Distribution of the number of tokens per patient trajectory, for the prediction of disease-free survival 5 years after surgery. 610 samples exceed the maximum sequence length for Tabular BEHRT (512 tokens). This represents an average of 10 visits per patient that are not considered by Tabular BEHRT.

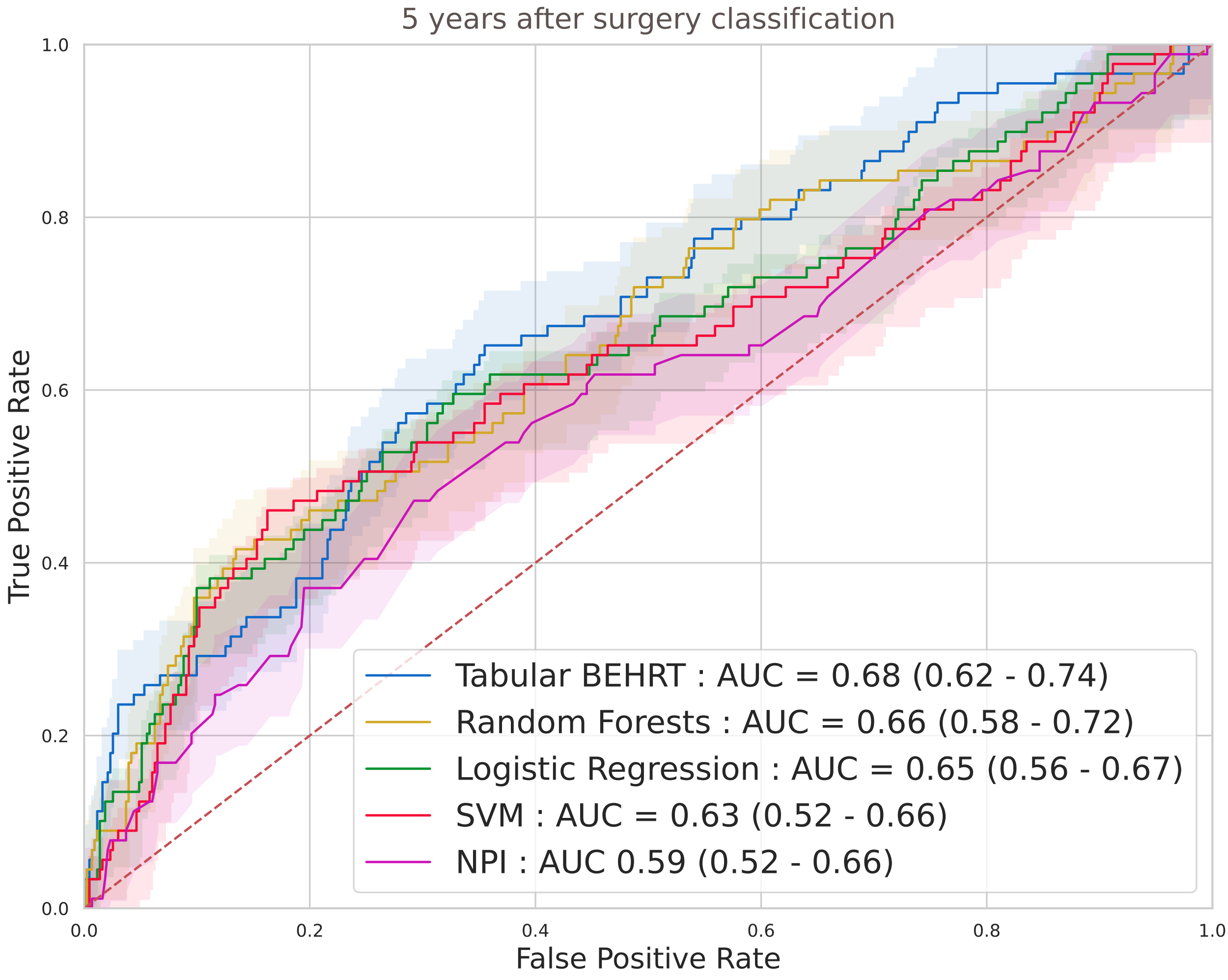

Figure S13. ROC curves for baselines and Tabular BEHRT, for predicting disease-free survival 5 years after surgery.

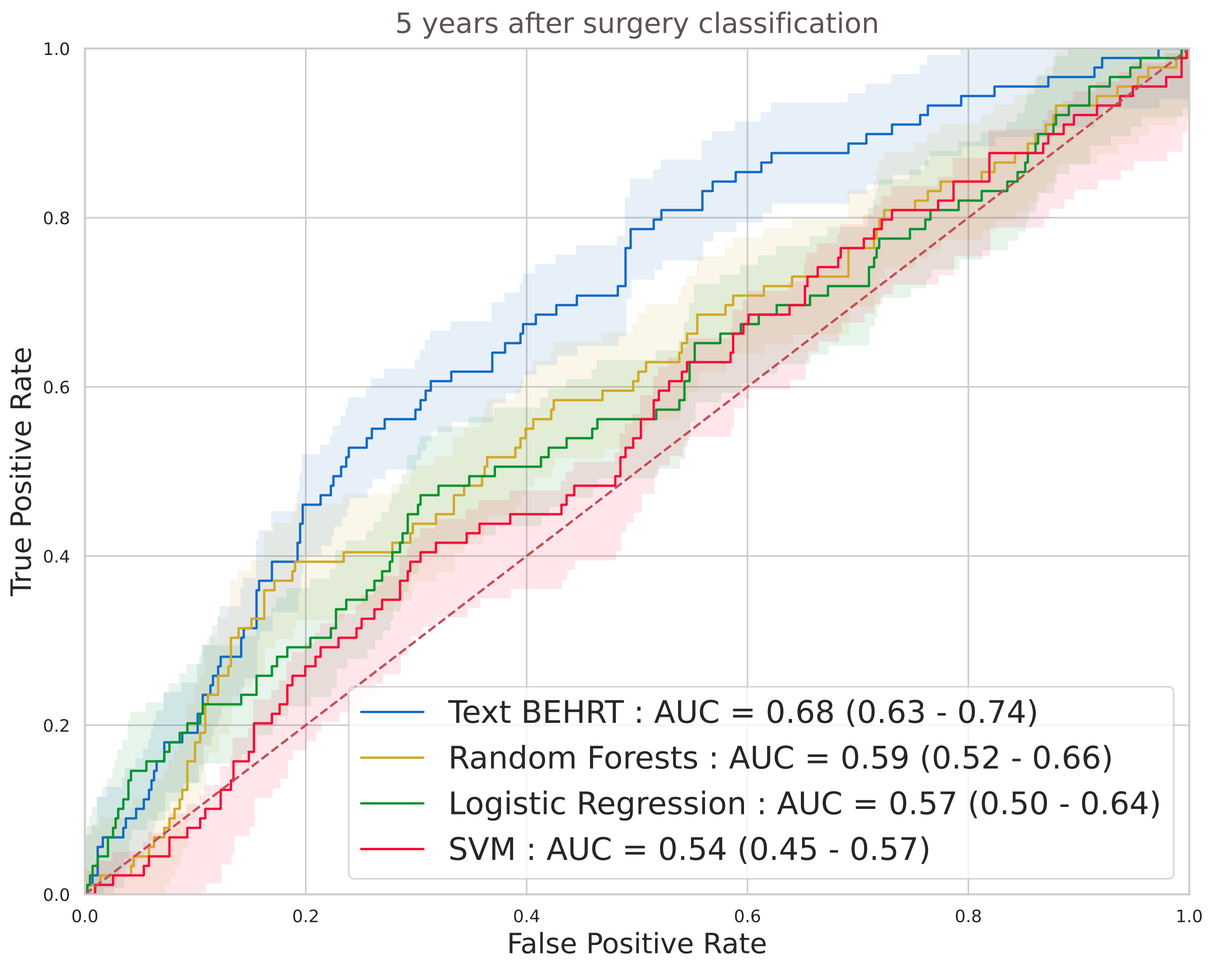

Figure S14. ROC curves for baselines and Text BEHRT, for predicting disease-free survival 5 years after surgery.

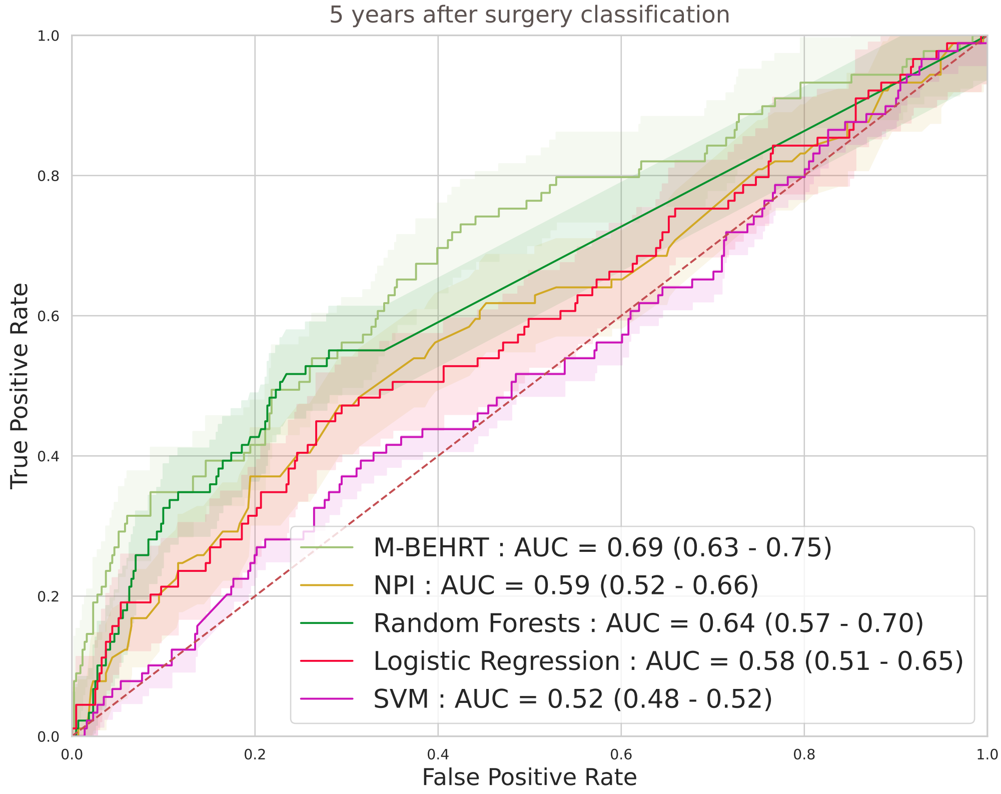

Figure S15. ROC curves M-BEHRT and baselines, for predicting disease-free survival 5 years after surgery.

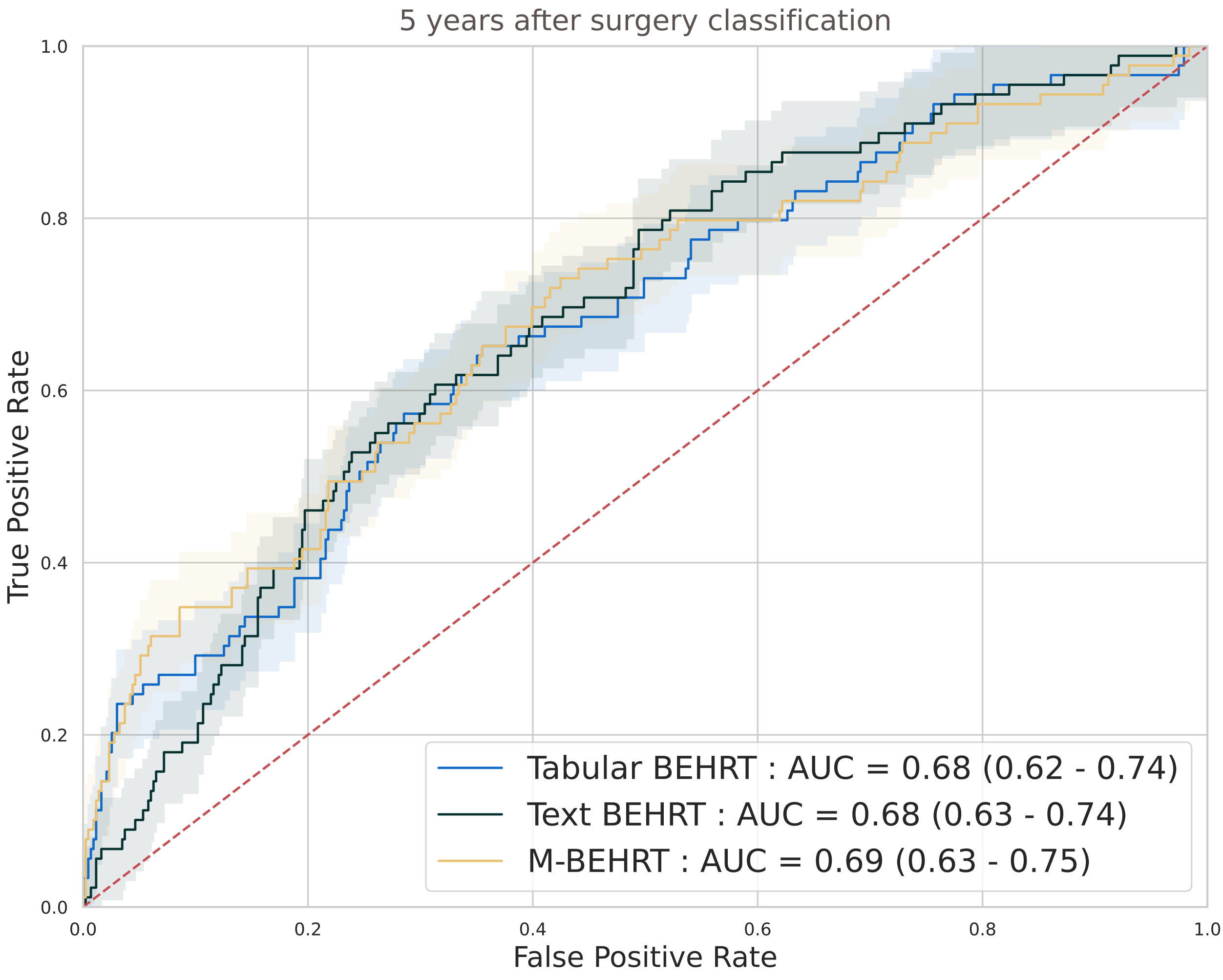

Figure S16. ROC curves comparing Tabular BEHRT and Text BEHRT against their combined model M-BEHRT, for the prediction of disease-free survival 5 years after surgery.

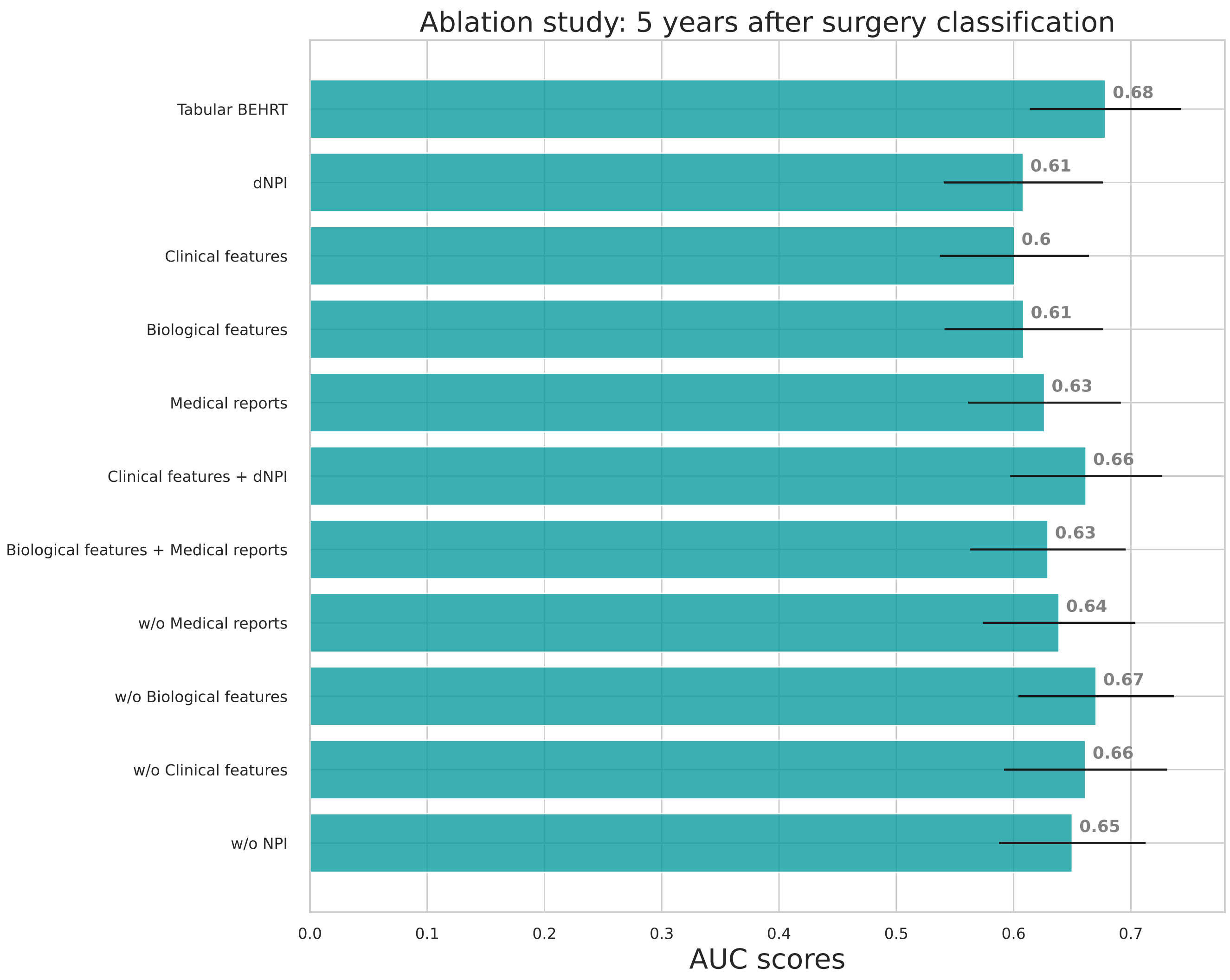

Figure S17. Ablation studies AUC-ROC on the test set for Tabular BEHRT, for the prediction of disease-free survival 5 years after surgery. We present results for the full model (Tabular BEHRT), then using only one of the 4 modalities (dNPI, clinical features, biological features, medical visits), two modalities (dNPI+clinical or biological+visits), then removing one of the 4 modalities. Here "medical records" stands for features extracted extracted from the medical record headers, that is to say, visit department and procedure. Performance scores are presented on the test set.

Variable	N	AUC score (95% Conf. Int.)
**Age**		
<50 (150:28)	178	0.63(0.53 to 0.74)
>=50 (281:61)	342	0.71(0.63 to 0.78)
**Molecular s/types**		
Luminal (232:50)	282	0.69(0.61 to 0.78)
Her2+ (74:12)	86	0.61(0.37 to 0.85)
Tnbc (44:9)	53	0.64(0.46 to 0.82)
**Nodes**		
N0 (310:54)	364	0.64(0.56 to 0.72)
N+ (121:35)	156	0.73(0.63 to 0.83)
**Grade**		
Grade i (75:9)	84	0.70(0.57 to 0.84)
Grade ii (184:35)	219	0.61(0.50 to 0.72)
Grade iii (172:45)	217	0.72(0.63 to 0.80)

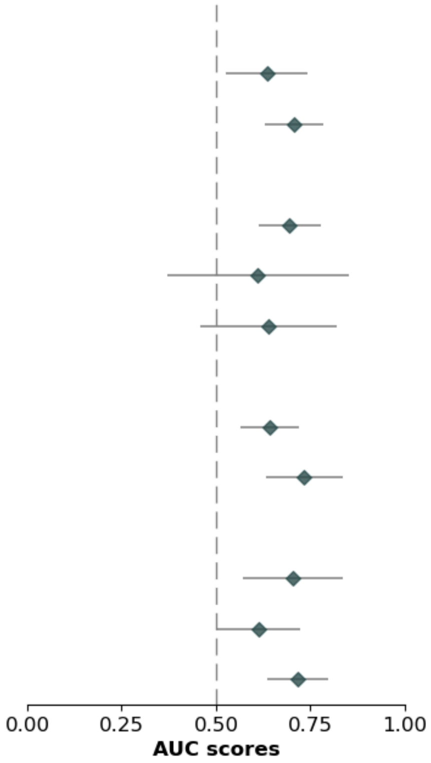

Figure S18. AUC-ROC of M-BEHRT stratified by patient age, cancer grade, molecular subtype and node status, for the prediction of disease-free survival 5 years after surgery.

Variable	N	AUC score (95% Conf. Int.)
**VPPG**		
DNPI >= 6.4 (10:7)	17	0.61(0.47 to 0.75)
**PPG**		
5.4 <= DNPI < 6.4 (31:11)	42	0.67(0.55 to 0.80)
**MPGI**		
4.4 <= DNPI < 5.4 (121:21)	142	0.62(0.48 to 0.76)
**MPGII**		
3.4 <= DNPI < 4.4 (71:18)	89	0.70(0.57 to 0.83)
**GPG**		
2.4 <= DNPI < 3.4 (137:21)	158	0.83(0.69 to 0.98)
**EPG**		
DNPI <=2.4 (61:11)	72	0.69(0.41 to 0.96)

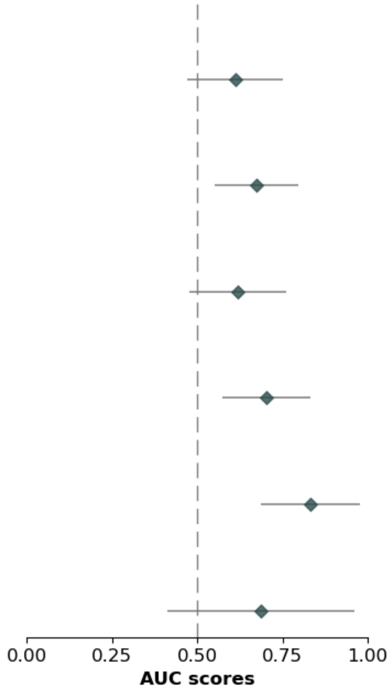

Figure S19. AUC-ROC of M-BEHRT stratified by NPI, for predicting disease-free survival 5 years after surgery.

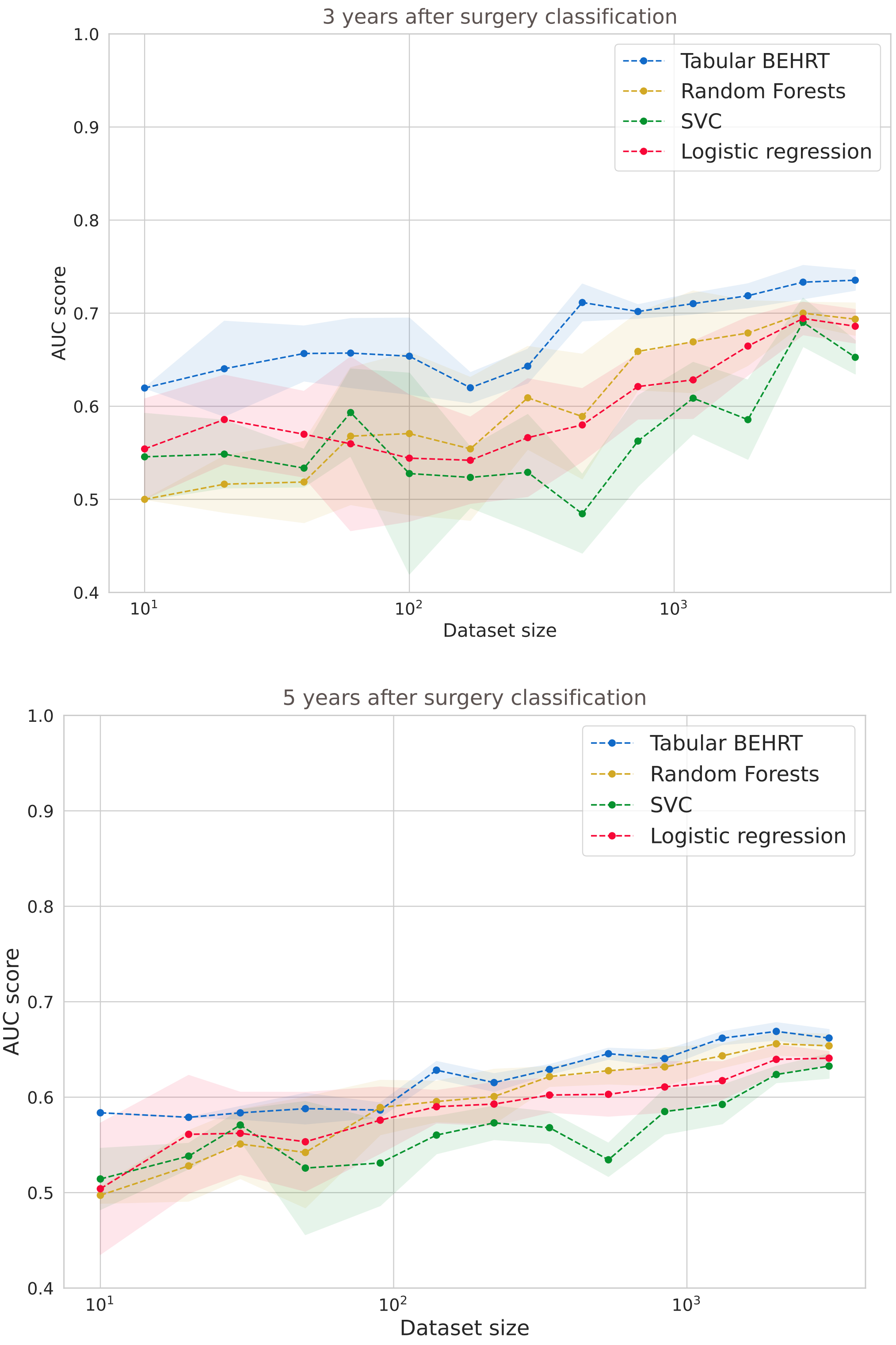

Figure S20. AUC-ROC on the test set of Tabular BEHRT, random forests, support vector classifier, and logistic regression trained on subsets of the training set of increasing sizes (x-axis), for the prediction of disease-free survival 3 (top) or 5 (bottom) years after surgery.
